# A Diagnosis of Turner Syndrome in the Eighth Decade of Life

**DOI:** 10.1210/jcemcr/luae104

**Published:** 2024-06-21

**Authors:** Ruveena Kaur, Susannah O’Sullivan

**Affiliations:** Department of Endocrinology, Greenlane Clinical Centre, Auckland 1051, New Zealand; Department of Endocrinology, Greenlane Clinical Centre, Auckland 1051, New Zealand

**Keywords:** Turner syndrome, delayed diagnosis, fracture risk, osteoporosis, hormone therapy

## Abstract

Turner syndrome (TS) is the most common sex chromosome disorder affecting females and is usually diagnosed within the first 3 decades of life. It can present with primary amenorrhea or infertility and often has a typical phenotype, with associated medical conditions that require lifelong surveillance. We report the case of a 76-year-old female with a history of osteoporosis and vertebral fractures who presented to our specialist osteoporosis clinic following a neck of femur fracture. She revealed a history of short stature and primary amenorrhea as a young woman, with limited investigation and treatment. Her other medical history included coeliac disease, hypertension, and hearing and vision abnormalities. Given her phenotype, the patient was referred for a karyotype at age 76, which was consistent with mosaic TS (45, X in 78% of cells and 46, X, r(Y) in the remaining cells). We review reports of other cases of marked delay in TS diagnosis and discuss the consequences of a late diagnosis.

## Introduction

Turner syndrome (TS) is a chromosomal disorder, characterized by distinct phenotypic features in a female with 1 intact X chromosome and complete or partial absence of the second X chromosome (1). The incidence is estimated at between 17 to 88 per 100 000 females, depending on the population and method of karyotype analysis (1). Typical features include short stature, hypergonadotropic hypogonadism, cardiovascular and renal abnormalities, hearing and visual impairment, and neurocognitive deficits (2). TS is also associated with an increased risk of metabolic complications and osteoporosis and a predisposition to autoimmunity. Mortality is increased at least 3-fold in these individuals, primarily from cardiovascular abnormalities (1, 3).

The diagnosis of TS is typically made by chromosomal analysis of at least 30 peripheral blood cells to identify about 10% mosaicism with 95% certainty (4). The most common chromosomal abnormality in TS patients is monosomy X (45, X), occurring in 40% to 50% of individuals (1). Other abnormalities include various forms of mosaicism such as 45, X/46, XX (15-25%), presence of a Y chromosome (including 45, X/46XY mosaicism) (10-12%), “Triple X” (45, X/47, XXX; 45, X/46XX/47, XXX) (3%), and an isochromosome Xq, isodicentric Xp [46, X i(Xq); 46, Xidic(Xp)] and ring X (10%) (1). There is no clear correlation between genotype and phenotype, although those with mosaicism tend to have a less severe phenotype (1).

Although the median age of TS diagnosis is 15 years, there are 3 main periods in life for diagnosis: in the neonate with abnormal findings on fetal ultrasound (5); aged 5 to 20 years while investigating for short stature, oligo/amenorrhea, or delayed puberty (1); and between age 30 and40 years with infertility as the presenting complaint. There is a wide range of clinical presentations, which often result in delayed diagnosis, particularly in patients with mosaic TS who can present atypically (1). This can have significant implications as therapy not initiated at the appropriate age can lead to inadequate surveillance and management of associated conditions.

This paper reports a patient who was only diagnosed with TS in her eighth decade of life, despite an initial presentation with primary amenorrhea and short stature and the development of several complications associated with TS.

## Case Presentation

A 76-year-old woman was assessed in a specialist osteoporosis clinic after sustaining a neck of femur fracture following a fall. She was known to have osteoporosis based upon atraumatic thoracic and lumbar fractures and a dual-energy x-ray absorptiometry (DXA) scan demonstrating bone mineral densities (BMD) in the osteoporotic range at the lumbar vertebrae. The patient had been treated with weekly alendronate 70 mg and monthly cholecalciferol 1.25 mg.

On inquiry, the patient revealed a past medical history of primary amenorrhea, which had been investigated by a gynecologist at age 12 years. She was treated with a combined oral contraceptive pill for a year, leading to modest breast development but no menstruation. She was also noted to be of short stature compared to other family members, but this was not inquired. At age 66 years, the patient was diagnosed with bilateral sensorineural hearing loss and cataracts. At age 70 years, she was diagnosed with coeliac disease by duodenal biopsy after presenting with gastrointestinal upset and weight loss and has since adhered to a gluten-free diet. At age 72 years, she was found to be markedly hypertensive during assessment for a potential kidney donation and commenced antihypertensive treatment with candesartan 16 mg and bisoprolol 2.5 mg daily.

The patient is a widow and has 2 adopted children. She was a bookkeeper and achieved an average academic standard during her school years. There is no family history of reproductive, cardiovascular, metabolic, autoimmune, or endocrine conditions.

## Diagnostic Assessment

On examination, the patient did not have the facial characteristics of TS. She was of short stature, with a height of 145.5 cm (mid-parental height 168 cm) and weight 67 kg, giving her a body mass index of 31.9 kg/m^2^. Her blood pressure was elevated at 180/80 mmHg with a wide pulse pressure. Examination revealed a broad-shaped chest with modest breast development (Tanner stage 3). She had normal heart sounds with no murmurs.

An updated DXA demonstrated a 21.4% increase in bone density at the lumbar spine (L1-L2 0.850 g/cm^2^, T-score −2.6), following 10 years of continuous alendronate therapy. At the right neck of femur, BMD was 0.795 g/cm^2^ and T score −1.8, and at the right total hip BMD was 0.795 g/cm^2^ and T score −1.7 (the left hip had been measured in her first DXA). In the 11 years between her 2 BMD measurements, she had lost 2.5 cm in height.

Laboratory investigations included a normal serum calcium and phosphate, with an estimated glomular filtration rate of 62 mL/minute. The bone formation marker procollagen 1 N-telopeptide measured 3 months following her hip fracture was 114 ug/L (reference range 20-115 ug/L). She was euthyroid with negative thyroid peroxidase antibodies and had a normal glycated hemoglobin and lipid profile.

Given her menstrual history and small stature, the gold standard peripheral blood karyotype including 30 cells was performed, which revealed a mosaic female karyotype with 2 cell lines: 18/30 cells (77.7% of cells) showed a female karyotype with 45, X, and in 12/30 cells (22.3% cells), 1 X chromosome in addition to a ring Y chromosome was classified as 46, X, r(Y). A subsequent fluorescence in situ hybridization analysis confirmed the presence of the *SRY* gene locus and Y chromosome centromeric region.

## Treatment

Considering her recent fracture and history of coeliac disease, a decision was made to cease the oral bisphosphonate and administer intravenous bisphosphonate (zoledronate 5 mg) in combination with monthly cholecalciferol. A further zoledronate infusion is planned in 18 months. To optimize her blood pressure management, bisoprolol was up-titrated to target a blood pressure of <140/90 mmHg.

## Outcome and Follow-up

Following her diagnosis, the patient underwent further screening investigations for conditions associated with TS. Abdominal ultrasound found bilateral, simple renal cysts, without evidence of a horseshoe appearance. A pelvic ultrasound was unable to identify the uterus or the left ovary. Magnetic resonance imaging pelvis demonstrated bilateral streak gonads with a markedly atrophic uterus, without evidence of gonadoblastoma. A transthoracic echocardiogram demonstrated a severely dilated left atrium, normal left ventricular size, and systolic function. The aortic valve was trileaflet with trivial aortic regurgitation. The aortic sinuses and proximal ascending aorta were within normal limits.

The patient will have an annual endocrinology review with screening for TS-related conditions as per international consensus guidelines (2). A repeat measurement of bone density is planned after the second zoledronate infusion.

## Discussion

This case report highlights the consequences of a delayed TS diagnosis leading to suboptimal treatment of estrogen deficiency and short stature, along with a failure to identify and manage conditions associated with TS. It is unclear whether a karyotype was performed when the patient first presented at age 12, but it is possible that an inadequate number of cells were sampled. This is a cited reason for delayed diagnosis in other individuals with TS (1). In the following years, the patient was diagnosed with and treated for conditions associated with TS including osteoporosis, sensorineural hearing loss, cataracts, coeliac disease, and hypertension. However, the possibility of TS was not considered until she was assessed by a reproductive endocrinologist.

Although most individuals with TS are diagnosed in their mid-teens, there remain instances of delayed diagnosis (and likely nondiagnosis). Howarth et al reported a 50-year-old newly identified patieint with TS during COVID after she received a shielding letter from the UK government (6). Originally diagnosed with TS as a neonate, she was subsequently lost to follow-up. At age 18 years she was investigated for delayed puberty and primary amenorrhea, with laparoscopic findings of a small uterus but was not initiated on hormone therapy (6). In addition to a genetic diagnosis of TS, she had comorbidities of hypertension, type 2 diabetes, recurrent otitis media, and reduced bone density (6). In Korea, a 61-year-old was diagnosed with TS after presenting with right-sided heart failure and an echocardiogram demonstrating bicuspid aortic valve with moderate aortic stenosis (7). Next-generation sequencing unexpectedly detected a Y chromosome, and chromosomal analysis was consistent with mosaic TS (7). In retrospect, she had premature ovarian insufficiency at age 38 years without initiating hormone treatment. Following her diagnosis of TS, she had a DXA demonstrating osteoporosis and commenced bone-protection treatment (7).

The delay in diagnosis of TS may result from a lack of syndrome recognition by clinicians, delayed presentation, and/or inadequate genetic analysis (1). Such a delay may have significant implications for individuals with TS. It can result in therapy not being initiated at age-appropriate stages, such as GH in childhood to achieve optimum height or estrogen for pubertal induction, bone acquisition, and uterine development. Subsequently, there may also be inadequate assessment, monitoring, and treatment of the conditions associated with TS. For our patient, this meant she was not diagnosed with osteoporosis until she sustained fractures. Individuals with TS with untreated estrogen deficiency are at increased risk of osteoporosis with a 25% increased risk of fracture (1). The induction of puberty with estrogen and its continued use until the natural age of menopause improves peak bone mass accrual and maintains bone density, with a younger age of puberty induction associated with higher peak BMD (8).

In the case of our patient, the delayed diagnosis of TS also prevented an adequate karyotype analysis. When this was eventually performed, she was found to have a ring Y chromosome, karyotype 45, X/46, X, r(y). It is essential that individuals with TS have an adequate assessment for the presence of a Y chromosome. This is important as an estimated 10% of individuals with TS and a detectable Y chromosome will develop a gonadoblastoma compared to 1% of all TS individuals (2). Current guidelines recommend all TS individuals with Y material undergo prophylactic gonadectomy (2). When our patient was investigated with pelvic magnetic resonance imaging, her gonads and uterus were markedly atrophic with no radiologic evidence of gonadoblastoma. Given her age and low probability of developing a malignancy, radiological surveillance was preferred over gonadectomy.

In conclusion, this case report highlights a marked delay in TS diagnosis and to our knowledge is the oldest age of documented TS diagnosis. It highlights the complications associated with inadequate hormone treatment and the consequences of a delay in the identification and appropriate management of associated comorbidities. TS is the most common sex chromosome related condition affecting girls and women and is an important condition for health practitioners to be aware of in cases of primary (and secondary) amenorrhea. Timely diagnosis, through ensuring karyotype analysis with an adequate number of cells, allows the induction of age-appropriate treatment, with beneficial effects on final height, pubertal induction, uterine development, and accrual of peak bone mass and institution of appropriate monitoring for TS-related conditions.

## Learning Points

- TS is the most common sex chromosome related condition affecting girls and women and is an important condition for health practitioners to be aware of in cases of primary (and secondary) amenorrhea.
- TS may present to a range of medical specialists and requires lifelong monitoring of the associated conditions.
- Commencing hormone therapy with continuous estrogen (and continuous or cyclical progestin) is recommended for pubertal development and to optimize bone accrual.
- The gold standard when performing karyotype analysis for TS is a minimum of 30 cells in a peripheral blood sample.
- Patients with 45, X/46, XY mosaicism have a 10% risk of gonadoblastoma.

## Data Availability

Original data generated and analyzed for this case report are included in this published article.
